# A Low-Cost Surge Current Detection Sensor with Predictive Lifetime Display Function for Maintenance of Surge Protective Devices

**DOI:** 10.3390/s20082310

**Published:** 2020-04-18

**Authors:** Youngjun Lee, Young Sam Lee

**Affiliations:** 1 Department of Electrical Engineering, 100 Inha-ro, Michuhol-gu, Inha University, Incheon 22212, Korea; yjl0717@empal.com; 2 Sungjin Techwin Co., Ltd., 62, Yuseong-daero 877 beon-gil, Yuseong-gu, Daejeon 34127, Korea

**Keywords:** surge current detection sensor, lightning strike counter, low-cost surge current detection sensor, surge protective device (SPD), maintenance of SPDs, predictive lifetime of SPD

## Abstract

In this study, a low-cost surge current detection sensor (SCDS) that can measure high current surges using a low-current toroidal coil is proposed for maintenance of a surge protective device (SPD). The proposed SCDS is designed to display the predicted lifetime of the SPD based on the magnitude of surge current and number of surges. In addition, a method for measuring high surge current using a toroidal coil that can usually measure only low current is proposed. A lightning strike counter consists of a microcontroller with a low-power liquid crystal display (LCD) driver, 3 VDC lithium battery, and signal conditioning circuit that converts amplitude information of the surge current into duration information of a negative pulse to facilitate processing in the microcontroller. In this paper, we propose a software algorithm that can calculate the remaining lifetime of SPD based on the amplitude and number of surge currents. There is also an option to select the capacity of the surge protective device and the number of phase lines, allowing it to assess the predicted lifetime for various types of Class II SPDs. The proposed SCDS is measured as 7.2 μA from the battery power consumption test, and the service life is calculated to be 11.1 years. It meets the International Standard IEC62561-6 test conditions of the lightning strike counter and is expected to be useful in the maintenance of SPDs and lightning protection systems.

## 1. Introduction

Surge protective devices (SPDs) for low-voltage power distribution systems are essential for the operation of electrical systems. SPDs installed in building power distribution systems and industrial applications are generally used to ensure continuous operation [1]. SPDs eliminate electrical surges or impulses by acting as a low impedance path that turns the transient voltage into a current and shunts it along the return path, usually ground. By design, an SPD often acts as a ‘self-sacrifice’ device. In other words, the primary purpose of an SPD is to remove harmful voltage spikes from an electrical system even in conditions potentially harmful to the SPD. SPDs should not stop operating even if the circuit breaker is activated, the nearby system is damaged, or if there is smoke that causes a physical hazard to humans [2]. A metal oxide varistor (MOV) is widely used in low-voltage power systems as a core component in surge protectors because of its technical advantages and low cost. However, an MOV carries the risk of thermal runaway at the end of its lifetime. Thermal runaway tends to result in electrical hazards, such as short circuits or explosions. Thus, the MOV is designed with a thermal fuse spring or thermal fuse to prevent thermal runaway [3,4,5,6,7,8,9,10].

In the test list of the International Standard IEC61643-11 for surge protective devices, the thermal stability test is important for investigating the potential combustion and explosion of SPDs at the end of the MOV lifetime. The lifetime of the MOV is assessed by the amplitude of the surge current, the amount of energy, and the number of surges. The transient surge current flowing into the SPD is usually measured using a current transformer (CT) or Rogowski coil. CTs require a high current detection range and high frequency characteristics to measure transient surge currents, and they can cost thousands of dollars. The Rogowski coil is significantly less expensive, costing several hundred dollars, but is still relatively expensive compared to an SPD. Typical single-phase and three-phase Class II $I_{n}$20-kA SPDs cost less than one hundred dollars [11,12,13,14,15,16,17,18]. For this reason, SPDs are rarely used with devices that can estimate lifetime. For maintenance, SPDs are typically replaced by electrical facility managers when they are thought to be at the end of their lifetime based on verification through a status indicator. A remote-control method can identify the signal output from the remote terminals of the SPD, but it is rarely used as the installation of additional communication modules presents a cost issue. Another option is a surge counter can be installed in the SPD [19,20]. If lightning current above the critical current level flows in, the count of the surge counter increases. However, this does not indicate the remaining lifetime of the SPD. At the end of the lifetime of the SPD, it can no longer protect electrical devices from surges because it is separated from the electrical distribution system. Therefore, a device that can measure the predicted lifetime of an SPD at a market-acceptable cost is necessary. In this study, a surge current detection sensor (SCDS) with a reasonable cost is proposed that can estimate the remaining lifetime of an SPD. The proposed SCDS consists of a low-cost current sensor using small wound toroidal cores composed of iron powder, a signal conditioning circuit for converting the amplitude of a surge to duration width of a negative pulse for easy processing in a low-voltage microcontroller, a 3 V coin-cell battery, and a low-cost liquid crystal display (LCD) to predict the lifetime of the SPD based on the magnitude of surge currents and number of surges. In this study, we propose a low-cost SCDS and a predictive lifetime software algorithm for estimating the SPD predictive lifetime for the maintenance of the SPD.

## 2. Materials and Methods

### 2.1. Mechanical Design

**Figure 1:**
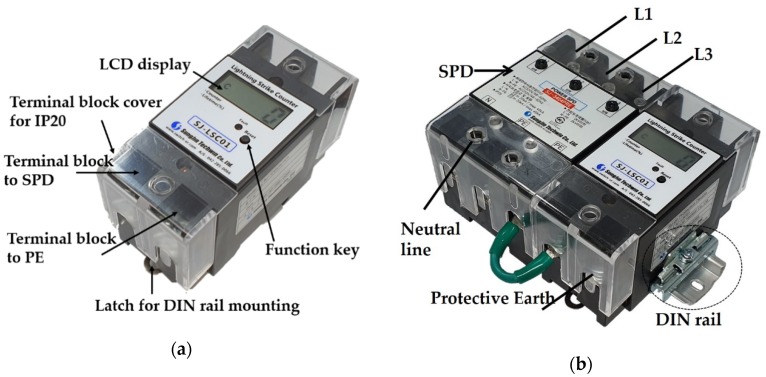
(**a**) Surge current detection sensor (**b**) Example of surge current detection sensor installed with a surge protective device (SPD).

The proposed SCDS is relatively small, with a size of 50.0 mm × 106.0 mm × 68.4 mm. The enclosure is composed of flame-retardant plastic and has a Protective Earth (PE) terminal connected to ground and a PE connection terminal connected to the SPD. For protection from electrical shock, the SCDS has a terminal block cover that meets the IP20 waterproof and dustproof rating. IP20 requires that the conductors of the SCDS are protected from human contact. The SCDS also has an LCD window and a function key on the top. For installation, it can be mounted on an EN 60715 DIN rail, which is the widely used mounting standard for electric devices such as circuit breakers. Figure 1a presents an external image of the SCDS; an image of the SCDS installed with the SPD is shown in Figure 1b.

### 2.2. Block Diagram

**Figure 2:**
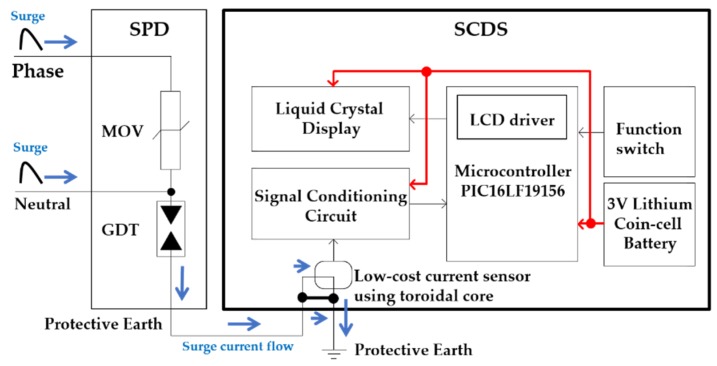
Block diagram of the proposed surge current detection sensor (SCDS).

Lightning causes transient surge voltage to the phase and neutral line of the SPD. The metal oxide varistor (MOV) and gas discharge tube (GDT) of the SPD limit the transient surge voltage and divert the surge current to PE. The surge current flows from the PE terminal of the SPD into PE through a low-cost current sensor using toroidal cores. The SCDS consists of the current sensor using toroidal cores with windings and a signal conditioning circuit for converting the amplitude of surge current to duration width of a negative pulse for microcontroller processing with a low operation voltage of 3 V. In addition, it uses a 3 V/1000 mAH coin-cell type battery for operation without an external power supply, and a low-cost liquid crystal display (LCD) to show the predictive lifetime of the SPD based on the amplitude of surge currents and the number of surges. Figure 2 shows the block diagram of the SCDS.

**Figure 3:**
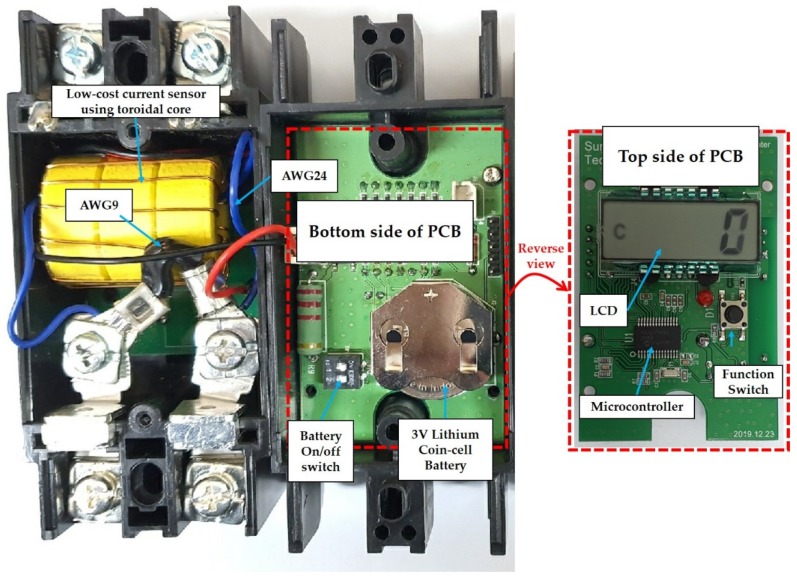
PCBs of the SCDS.

The interior consists of a current sensor PCB and a main PCB. The installed PCB is shown in Figure 3.

### 2.3. Lightning Surge

#### 2.3.1. Classification of Surges

**Figure 4:**
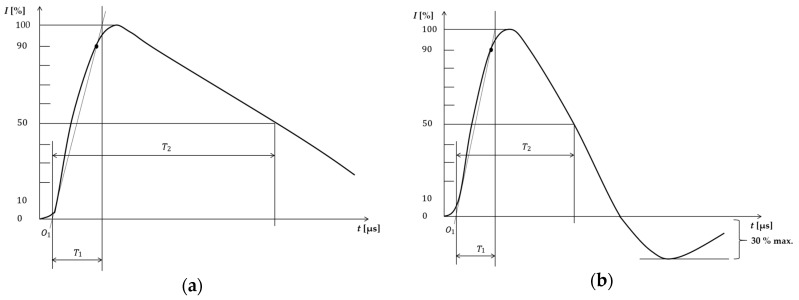
(**a**) Direct lightning surge current waveform (**b**) Induced lightning surge current waveform.

Surge transient voltages are caused by the switching operation (on/off) of electrical loads used in a building, and sometimes by magnetic and inductive coupling due to the formation of magnetic fields as large currents flow. Surges are also caused by static electricity and lightning strikes, which are classified as direct and induced lightning strikes. Direct lighting strikes travel directly through lightning rods or antennas and can damage electronic devices through high energy impulse-induced strike currents. When a direct lightning strike hits a line, the surge current is divided into two parts by the flow direction. The surge voltage is determined by the surge impedance of the line and the surge current [12].

(1) $$V=\frac{Z\times I}{2}$$

where V is the surge voltage, Z is the surge impedance of the line, and I is the surge current. Assuming a surge current of 10 kA and a surge impedance of 400 Ω, the surge voltage is calculated to be 2000 kV by Equation (1). Owing to changes in the electromagnetic field that occur during lightning strikes, surges can also occur on power lines that are far from the lightning strike. The surge voltage of the induced lightning can be obtained from [12]

(2) $$V=30\times k\times \left(\frac{h}{d}\right)\times I$$

where V is the prospective surge voltage, I is the lightning current, h is the height above the ground of the conductors, k is a factor depending on the velocity of the return stroke of the lightning strike path, and d is the distance away from the lightning strike. The value of k generally lies in the range of 1.0–1.3. For example, the surge voltage caused by a lightning strike at a distance of 1 km under a surge current of 10 kA and a line height of 5 m is calculated to be 1.5 kV. High surge voltage is also generated by an induced lightning strike. A direct lightning current may amount to hundreds of kA, which can cause damage from induced lightning strikes at a distance of tens of km. Damage to electronic devices caused by lightning strikes is usually from induced lightning. Therefore, to prevent damage caused by lightning strikes, an SPD is used as indoor lightning protection in buildings [12]. The international standard waveforms of lightning surge current for direct and induced lightning strikes are depicted in Figure 4, and associated parameters are presented in Table 1 [11,12].

Here, $O_{1}$ is the virtual origin, I is the peak current, $T_{1}$ is the front time, and $T_{2}$ is the time to half value. $I_{imp}$ is the impulse discharge current, the SPD capacity of Class I that can withstand a 10/350 μs surge current flowing through the SPD. $I_{n}$ is the nominal discharge current, which is the SPD capacity of Class II that can withstand an 8/20 μs surge current flowing through the SPD.

#### 2.3.2. Lifetime of MOV

**Figure 5:**
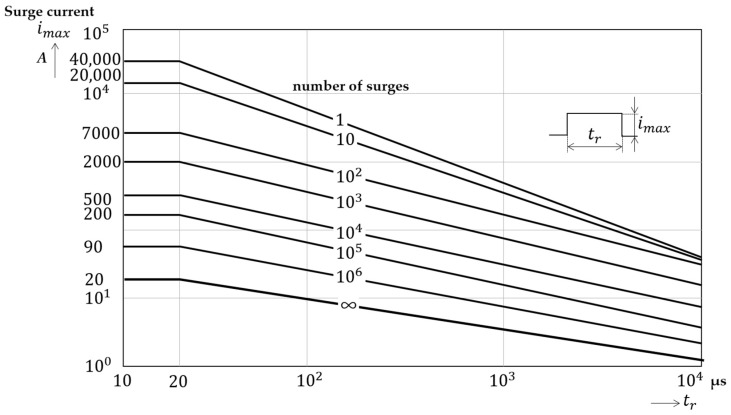
Derating curves of $I_{n}$ 20 kA metal oxide varistor (MOV).

The derating curves of the $I_{n}$ 20 kA MOV (manufactured by TDK Electronics) used as a core component in Class II SPDs are presented in Figure 5 [21], where $I_{n}$ is an 8/20 μs current that flows through the SPD and is the nominal discharge current for a Class II SPD. Figure 5 shows that the number of endurable surges is determined by the amplitude of the surges and their duration time. For a duration time of 20 μs, the rated number of surges exponentially increases as the amplitude of the surge current decreases. The lifetime of the MOV can be calculated based on the amplitude and number of surge currents.

### 2.4. Toroidal Coil Type Current Sensor and Signal Conditioning Circuit Design

#### 2.4.1. Toroidal Coil Type Current Sensor

**Figure 6:**
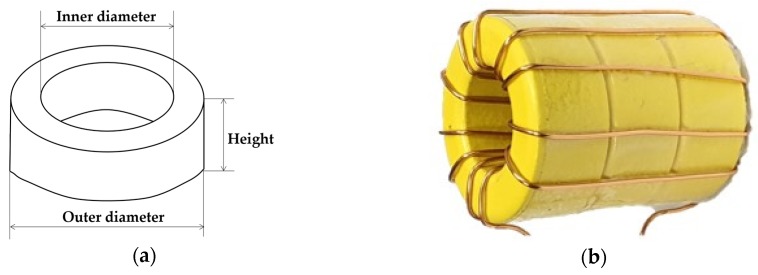
(**a**) Shape of toroidal core (**b**) Toroidal current sensor.

Generally, surge current is detected using a current transformer (CT). To measure high surge current, a Rogowski coil or a large current transformer (CT) with good frequency characteristics is used. However, CTs are costly and not economical. Thus, three inexpensive toroidal cores (Part No. C27-B11) from Core Electronics composed of ordinary iron powder are used [22]. The toroidal current sensor with a ∅1.2 winding of 11 turns around the toroidal core is shown in Figure 6. The material and dimensions of the toroidal core are presented in Table 2.

#### 2.4.2. Signal Conditioning Circuit

**Figure 7:**
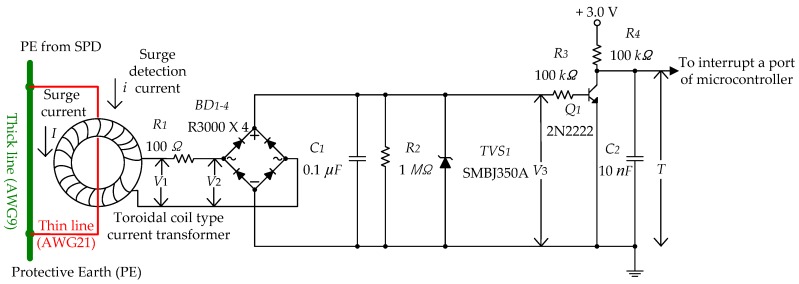
Signal conditioning circuit for detecting the surge current.

The signal conditioning circuit used for the detection of the surge current prevents magnetic saturation of the toroidal core and converts the amplitude of the surge current to the duration width of a negative pulse. The signal conditioning circuit diagram is shown in Figure 7.

The voltage $V_{1}$ induced on the secondary side by the surge current *I* to the PE on the primary side of the toroidal coil is proportional to the time derivative of the magnetic flux.

(3) $$V_{1}=-nA\times \frac{\partial B}{\partial t}$$

where *n* is the number of secondary windings, *A* is the area of the core’s cross section, and *B* is the magnetic flux, which is proportional to surge current *I*. Generally, iron toroidal coils are not used to measure high-surge current due to magnetic saturation. However, in Figure 7, the PE terminals are directly connected as shown by the thick line (American Wire Gauge, AWG9), and the thin line (AWG21) is connected through the center of the toroidal coil to prevent magnetic saturation. Magnetic saturation is prevented because the direct connection depicted by the thick line carries high current, and the connection depicted by the thin line through the toroidal coil carries low current. The resistance value of AWG21 is approximately 16 times higher than that of AWG9. Moreover, the cable length of AWG21 is twice the length of AWG9. The resistance can be calculated as [23]

(4) $$R=\rho \frac{l}{A},$$

where R is the resistance, ρ is the resistivity, *A* is the area of the cross section, and l is the length.

The current flowing in the wire is governed by Kirchhoff’s Current Law [24]. Thus, magnetic saturation of the toroidal core with high current surges is prevented. The area of the cross section, resistance, and current distribution rate according to cable length are shown in Table 3. The proposed method can withstand current up to 30 times the maximum current that can be measured by the toroidal core.

**Figure 8:**
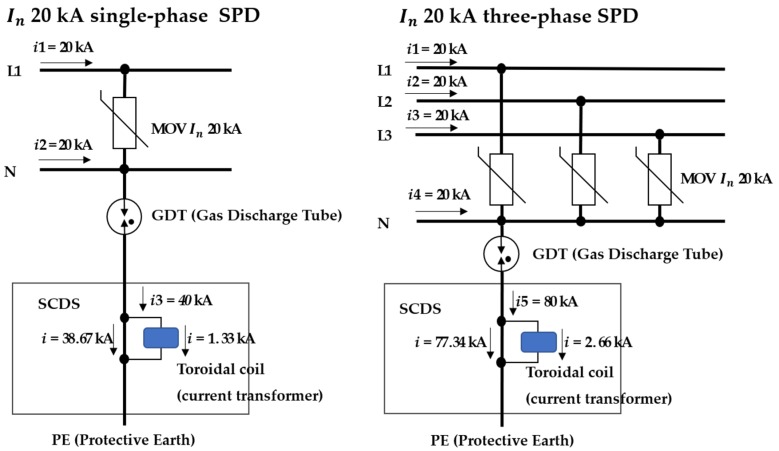
Example circuit for maximum surge current that flows through the PE path.

In the International Standard IEC61643-11, the maximum surge currents allowed in lines of the SPD in Class I and Class II are $I_{imp}$ 25 kA and $I_{n}$ 20 kA, respectively. The currents of *I* and *i* divided from the total surge current flowing through the path of PE are presented in Table 4, and examples of the current distribution according to single-phase and three-phase power are illustrated in Figure 8 [11,12].

**Figure 9:**
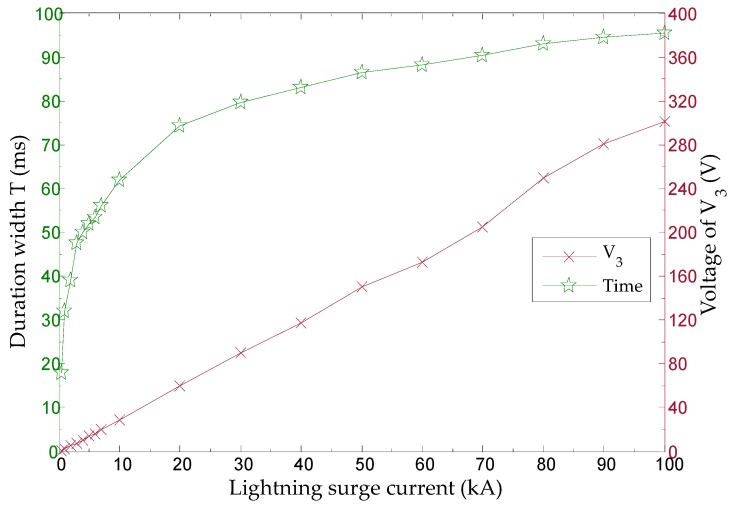
Output characteristics of signal conditioning circuit.

The magnetic saturation threshold of the surge current (8/20 μs) flowing through the toroidal coil is 3.3 kA, as measured by an experiment. Thus, a current of up to 100 kA can be measured by the SCDS. Because the output voltage may be high proportional to the current, the $V_{2}$ voltage is reduced by connecting $R_{1}$ (100 Ω) in series with the output of the toroidal coil. The bridge diode ($BD_{1}$) is used to detect both positive and negative surge voltages using the R3000 from RECTRON Semiconductor. Considering the high surge voltage input, a component with the maximum repetitive peak reverse voltage is chosen. The peak voltage of the surge is charged through $C_{1}$ and discharged through $R_{2}$ and $R_{3}$—there is no other discharge path. The output of the signal conditioning circuit is a negative pulse whose duration width is proportional to the amplitude of the surge current. $TVS_{1}$ is used to protect the $Q_{1}$ 2N2222 NPN transistor. The collector of $Q_{1}$ yields 3 V, recognized as logic high in normal conditions. The voltage of $V_{3}$ is logic low when it is above the threshold voltage of $Q_{1}$ (typically 0.7 V). $R_{4}$ is a pull up resistor, and $C_{2}$ is used to reduce noise and chattering. Let $V_{3,peak}$ be the peak value of $V_{3}$. Then, we have the following relationship for the collector output of $Q_{1}$:

(5) $$T=\frac{R_{2}R_{3}}{R_{2}+R_{3}}C_{1}\times \ln(\frac{V_{3,peak}}{V_{th}})$$

where *T* is duration width (surge detection interrupt time) of the logic low state of a negative pulse used as an input to the microcontroller, $R_{2}$ is 1 MΩ, $R_{3}$ is 100 kΩ, $C_{1}$ is 0.1 μF, $V_{3,peak}$ is the peak voltage charged to $C_{1}$, and $V_{th}$ is the cut-off voltage between the base and the emitter of the $Q_{1}$ 2N2222 NPN transistor and is approximately 0.2 V. $V_{1,peak}$ and $V_{3,peak}$ of the current transformer (toroidal coil) according to the amplitude of the surge current, and the time duration T were measured through an experiment and are shown in Table 5. Figure 9 illustrates the output characteristics of the signal conditioning circuit.

**Figure 10:**
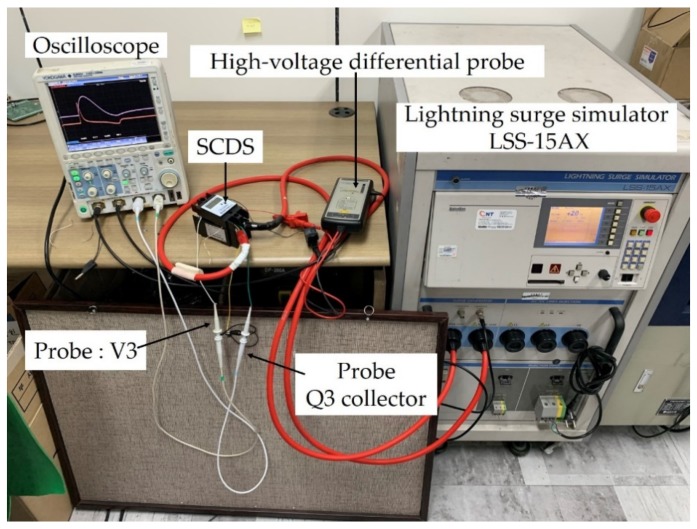
Experimental test setup for secondary output of toroidal coil type current transformer of SCDS.

The output of the current transformer (toroidal coil) due to the surge current is proportional to the time derivative of the surge current, as shown in Equation (3). The test setup is shown in Figure 10.

**Figure 11:**
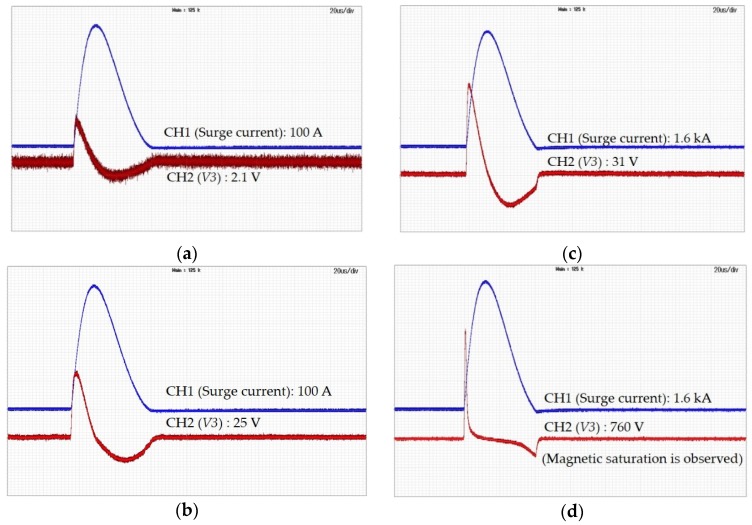
(**a**) Secondary output characteristics of a toroidal coil type current sensor with three cores and current distribution using AWG9 and AWG24 wires when the surge current was 100 A. No magnetic saturation was observed; (**b**) Secondary output characteristics of a toroidal coil type current sensor with three cores and current distribution using AWG9 and AWG24 wires when the surge current was 1.6 kA. No magnetic saturation was observed; (**c**) Secondary output characteristics of a toroidal coil type current sensor that used one core and did not distribute current when the surge current was 100 A. No magnetic saturation was observed; (**d**) Secondary output characteristics of a toroidal coil type current sensor that used one core and did not distribute current when the surge current is 1.6 kA. Magnetic saturation was observed.

The output waveforms measured using three cores and distribution of current using the AWG9 and AWG24 cables showed no magnetic saturation at 100 A and 1.6 kA, as shown in Figure 11a,b. The output waveform measured using one core and without current distribution does not show magnetic saturation at 100 A, as shown in Figure 11c, but magnetic saturation was observed at 1.6 kA, as shown in Figure 11d.

The test equipment used in this experiment is shown in Table 6. As the secondary side of the toroidal coil has a different reference point from the ground of the signal conditioning circuit, it was measured using a high-voltage differential probe [25,26].

**Figure 12:**
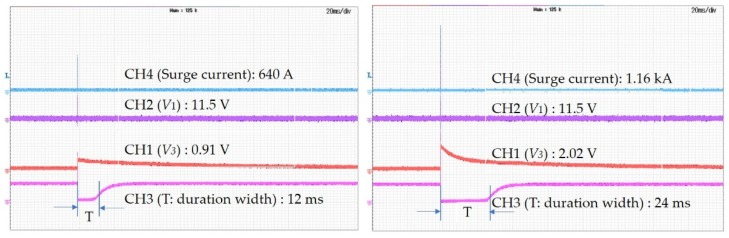
Amplitude of surge current and duration width of negative pulse generated by signal conditioning circuit.

Figure 12 illustrates the manner by which the amplitude information of the surge current is translated to the duration width of a negative pulse generated by the signal conditioning circuit. The amplitude of voltage $V_{3}$ is converted to T. As the $V_{3}$ voltage amplitude increases, the converted T increases, as shown in Equation (4), making processing easier in the microcontroller.

### 2.5. Calculation Principle of the Predictive Lifetime and Software Algorithm for the Expected Lifetime of the SPD

#### 2.5.1. Calculation Principle of the Predictive Lifetime of the SPD

**Figure 13:**
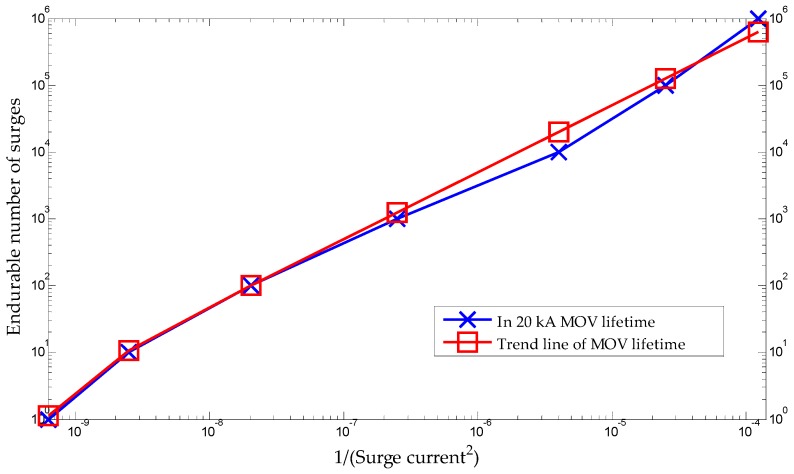
Trend line of MOV lifetime according to surge current and number of surges.

The predictive lifetime of the SPD using the SCDS proposed in this study can be calculated based on the lifetime of the MOV. The lifetime in the derating curves of the MOV decreases in proportion to the square of the current, as shown in Table 7. It can be expressed as a trend line, as shown in Figure 13.

The equation of the trend line of the MOV lifetime can be represented as

(6) N=αx−β

where x is the inverse of the square of current applied to the MOV, and *N* is the endurable number of surges. The constants α and β were found to be 5,012,836,137 and 2, respectively. Therefore, Equation (6) can be rewritten as

(7) $$N=\alpha \left(\frac{1}{{i_{mov}}^{2}}\right)-\beta$$

The calculation principle for the predictive lifetime of the SPD in the proposed SCDS can be expressed as

(8) $$L_{n+1}=L_{n}-\frac{1}{N}$$

where $L_{n+1}$ is the estimated value of the SPD lifetime after the surge input, $L_{n}$ is the SPD lifetime value before the surge input, and the initial value of $L_{n}$ is 1 ($L_{0}=1)$. Furthermore, $\frac{1}{N}$ is the decrease in the lifetime of the MOV according to the surge input, where *N* is determined from Equation (7). By substituting Equation (7) into Equation (8), the equation can be written as

(9) $$L_{n+1}=L_{n}-\frac{1}{\alpha \left(\frac{1}{{i_{mov}}^{2}}\right)-\beta }$$

$i_{mov}$ can be expressed as Equation (10), depending on the number of phase lines.

(10) $$i_{mov}=\frac{I_{total}}{P_{n}}$$

where $I_{total}$ is the total surge current flowing from all phase lines to the PE path, and $P_{n}$ is the total number of phase lines.

The final equation for the predictive lifetime can be expressed as

(11) $$L_{n+1}=L_{n}-\frac{1}{\alpha \left(\frac{{P_{n}}^{2}}{{I_{total}}^{2}}\right)-\beta }$$

#### 2.5.2. Software Algorithm for the Predictive Lifetime of the SPD

**Figure 14:**
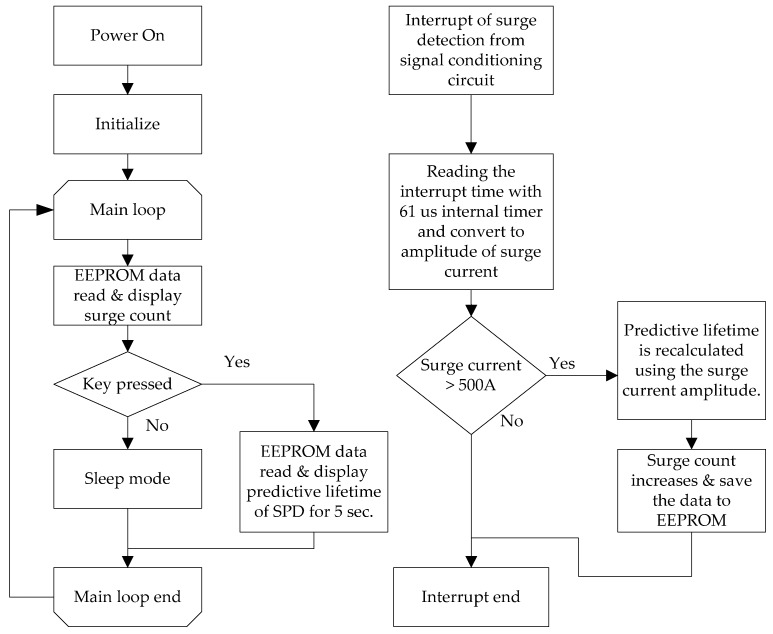
Software algorithm for the predictive lifetime of the SPD.

As power is applied to the SCDS, the microcontroller’s timer, interrupt, and embedded electrically erasable programmable read-only memory (EEPROM) are initialized. The surge count data of the EEPROM is read and displayed on the LCD, and sleep mode is activated to minimize battery power consumption. When the function key is pressed, the microcontroller exits sleep mode and displays the predictive lifetime of the SPD for five seconds. The surge count is displayed again, and the microcontroller returns to sleep mode. If surge interrupt is detected as ‘low (0 V)’ from the signal conditioning circuit in the main loop, the ‘low (0 V)’ holding time is measured by the internal 61-μs timer (2/clock: 32.768 kHz) and translated to the amplitude of the surge current. If the surge amplitude is below 500 A, it will be ignored, according to Table 8. If the surge current is above 500 A, the predictive lifetime of the SPD will be recalculated using the surge current amplitude. The number of surges and predictive lifetime are stored on the internal EEPROM. The increased surge count is displayed on the LCD. The software algorithm for the predictive lifetime of the SPD is shown in Figure 14.

**Figure 15:**
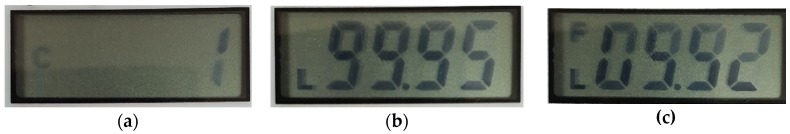
(**a**) Surge count mode (**b**) Lifetime mode (**c**) End of lifetime mode.

The proposed SCDS displays the surge count on the LCD in normal conditions. If a surge current is detected or the function button is pressed briefly, the lifetime is displayed for five seconds and the surge count is displayed. The display windows are designed as shown in Figure 15.

Figure 15a shows the surge count mode; ‘C’ on the left side indicates count mode. Figure 15b shows the lifetime mode; ‘L’ on the left side indicates lifetime mode. If the lifetime is less than 10%, ‘F’ in the upper left corner will be displayed, indicating the end of lifetime, as shown in Figure 15c.

**Figure 16:**
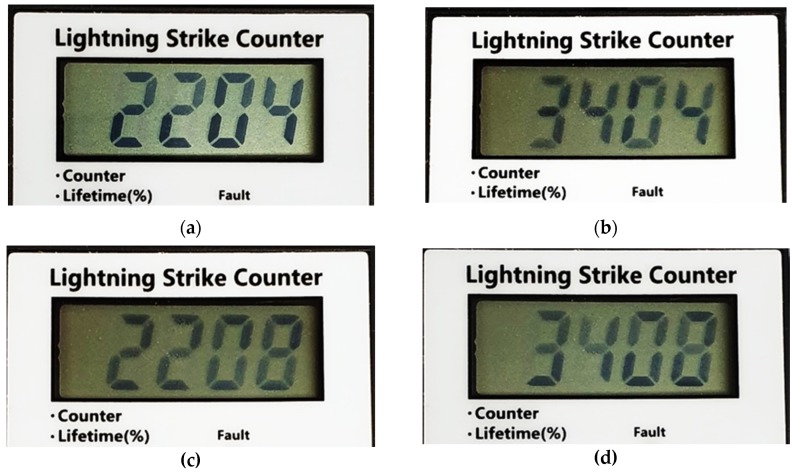
(**a**) $I_{n}$ 20-kA single-phase SPD selection mode (**b**) $I_{n}$ 20-kA three-phase SPD selection mode (**c**) $I_{n}$ 40-kA single-phase SPD selection mode (**d**) $I_{n}$ 40-kA three-phase SPD selection mode.

The proposed SCDS is designed to be used with various types of SPD. When the function key of the SCDS is pressed for more than ten seconds, it activates the SPD selection mode. A short press of the function key changes the SPD mode and pressing for more than three seconds selects it. The SPD selection mode is shown in Figure 16. The four numbers shown on the LCD are SPD model names. The first two numbers indicate single-phase (22) or three-phase (34) operation. The last two numbers indicate the capacity of the SPD; 04 indicates 20-kA capacity, and 08 indicates 40-kA capacity.

## 3. Results

### 3.1. Expected Lifetime of the SPD

#### IEC62561-6 Test Results

The IEC62561-6 international standard specifies requirements and tests for devices that count surges. Surge counters can be installed and used on the conductor of the LPS (lightning protection system) or the SPD. According to this standard, the operating threshold current $I_{tc}$ of the surge counter is defined as 500 A when used with the SPD and 1 kA when used with the LPS. The surge counter should not operate at half value of $I_{tc}$. $I_{mcw}$ is the maximum surge current that the surge counter can withstand. Typical values are shown in Table 8 [27].

In the proposed SCDS, the value of $I_{tc}$ was designed to be 500 A, according to Table 8. LSS-15AX (15 kV, 7.5 kA) by Noiseken was used for generation of surges. The results of the experiment meet the requirements of IEC62561-6, as shown in Table 9 [27].

$I_{mcw}$ was also tested by the National Accredited Certification Body and the SSG Series from HAEFELY was used for generation of the impulse current. Operation was normal with no damage when the tests were performed by applying 8/20 μs 100 kA surge current to the SCDS. Surge currents of both polarities were tested.

### 3.2. Results of the Predictive Lifetime Test of the SPD

To test the predictive lifetime of the SPD, the SCDS, configured as a three-phase four-wire system, was connected, and the lifetime reduction test was performed by an authorized institution using an impulse current generator (SSG Series) to vary the current amplitude. The test results are shown in Table 10, with error tolerances within 10%.

### 3.3. Battery Power Consumption Test Results

**Figure 17:**
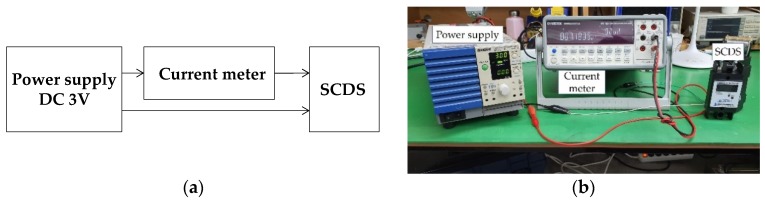
(**a**) Battery consumption test setup (**b**) Battery consumption test configuration.

The proposed SCDS is powered by a built-in 3 V coin-cell type battery without an external power supply, so it can be operated at ultra-low power for a long period of time. For the power consumption test, a PAS40-18 (40 V/18 A) from KIKUSHI Electronics Corp (Kanagawa County, Japan). was used as a power supply, and a GDM-8261A by GWINSTEK (Xinbei, Taiwan) was used as an ampere-meter. Figure 17 shows the test setup.

After applying 3 VDC from the power supply, the measured current in sleep mode was approximately 7.2 μA and the measured current in wakeup mode was approximately 100 μA. The wakeup mode period is 75 ms at 20 kA. Assuming that the SCDS is detected 100 times at 20 kA, it is 7.5 s, so it can be regarded as not affecting the power consumption of the battery. And the battery life was calculated to be 11.1 years, as shown in Equation (12) [28]. Thus, the target lifetime of seven years (typically the maximum lifetime of an SPD) is guaranteed.

(12) $$Battery\;Life=\frac{Battery\;Capacity\;\left(mAH\right)}{Current\;Consumption\;\left(mA\right)}\times 0.7÷\left(24\;\left(hour\right)\times 365\left(day\right)\right)≅11.10\;years$$

In Equation (12), a constant of 0.7 is used as a margin factor to consider environmental conditions of humidity and temperature.

## 4. Discussion

It has been confirmed that the proposed SCDS operates according to the amplitude of lightning surge currents and has a current detection error tolerance of approximately 10%. In practical applications, tolerances near 10% are not particularly critical. Currently, lifetime indicators are rarely used owing to relatively high cost compared to the SPD, and low-cost surge counters are used instead. The surge counters count detections of current above the threshold surge current, but do not indicate the lifetime of the SPD. Table 11 shows a comparison of performance, cost, and market applicability for the proposed SCDS with commonly used lightning counters and other SCDSs.

## 5. Conclusions

In this study, we proposed a low-cost surge current detection sensor, which can predict lifetime for maintaining surge protective devices by detecting the amplitude of surge currents and number of surges. The proposed SCDS improves on conventional surge counters that display only the number of surges. The proposed SCDS uses a low-cost toroidal coil to measure the surge current, and a new measurement method to solve the problem of magnetic saturation caused by high surge current. In addition, we proposed both a method and a software algorithm for calculating the predictive lifetime of an SPD in a microcontroller through a signal conditioning circuit that converts the amplitude of the surge current into the duration width of a negative pulse. The proposed SCDS is expected to greatly improve the maintenance efficiency of SPDs. In future research, we plan to study the predictive lifetime of a communication SPD.

## Figures and Tables

**Table 1:** Parameters of lightning surge current waveforms.

Lightning Surge Current	*T1* [μs]	*T2* [μs]	SPD Class	SPD Capacity
Direct	10	350	class I	$I_{imp}$
Induced	8	20	class II	$I_{n}$

**Table 2:** Dimensions and material characteristics of three toroidal cores.

Part No.	Inner Diameter	Outer Diameter	Height	Material	Frequency Range
C27-B11	14.48 mm	26.92 mm	11.1 × 3 mm	Iron powder and small other mixtures	500 kHz

**Table 3:** American Wire Gauge (AWG) 9 and AWG24 characteristics.

AWG	Cross-Sectional Area (mm^2^)	Resistance (Ω/m)	Cable Length (mm)	Connection Resistance (Ω)	Current Distribution Rate (%)
9	6.63	0.0026	30	0.001	≅97
24	0.205	0.0842	60	0.029	≅3

**Table 4:** Maximum Surge current values according to single-phase and three-phase power that flow through the Protective Earth (PE) path.

Description	Number of Power Lines	Maximum Surge Current (Induced, 8/20 μs, *I_n_* 20 kA)	Maximum Surge Current (Direct, 10/350 μs, *I_imp_* 25 kA)
Single-phase	2 (line, neutral): *i3*	Total 40 kA	Total 50 kA
	*I*	38.67 kA	48.34 kA
	*i*	1.33 kA	1.66 kA
Three-phase	4 (R, S, T, neutral): *i5*	Total 80 kA	Total 100 kA
	*I*	77.34 kA	96.67 kA
	*i*	2.66 kA	3.33 kA

**Table 5:** Experimental data of $V_{1,peak}$, $V_{3,peak}$, and T for different amplitudes of surge current.

Lightning Surge Current (kA)	T (ms)	$V_{1,peak}\;(V)$	$V_{3,peak}\;(V)$
0.25	5.29	5.4	0.3
0.5	18.00	11.1	0.85
1	32.00	22	2.24
2	39.00	42.2	5.24
3	47.50	66.2	7.7
4	50.00	90	10.6
5	52.00	130	13.7
6	53.50	151	16.2
7	56.00	175	19.6
10	62.00	235	28.6
20	74.09	435	58.6
30	79.48	635	88.6
40	83.29	835	118.6
50	86.23	1035	148.6
60	88.63	1235	178.6
70	90.65	1435	208.6
80	92.40	1635	238.6
90	93.95	1835	268.6
100	95.46	2035	301.6

**Table 6:** Test equipment to measure toroidal coil output and signal conditioning circuit of SCDS.

Test Equipment	Maker	Model	Specification
Lightning surge simulator	Noiseken	LSS-15AX	*Voc* (1.2/50 μs): 15 kV *Isc* (8/20 μs): 7.5 kA
Impulse current generator	HAEFELY	SSG series	*Isc* (8/20 μs): 120 kA
High voltage differential probe	Sapphire Instruments	SI-9010	±7000 V
Oscilloscope	Yokogawa	DLM2054	2.5 Gs, 500 MHz
Scope probe	Yokogawa	701939	600 V, 600 MHz

**Table 7:** $I_{n}$ 20 kA MOV lifetime characteristics according to the surge current and number of surges.

$i_{mov}$ Surge Current (A)	$\frac{1}{{\left(\mathrm{Surge Current}\right)}^{2}}$	Endurable Number of Surges
90	1.235 × 10^−4^	1,000,000
200	2.500 × 10^−5^	100,000
500	4.000 × 10^−6^	10,000
2000	2.500 × 10^−7^	1000
7000	2.041 × 10^−8^	100
20,000	2.500 × 10^−9^	10
40,000	6.250 × 10^−10^	1

**Table 8:** Typical values of $I_{tc}$ and $I_{mcw}$ for IEC62561-6.

Application	$I_{tc}$ Value		$I_{mcw}$ Value				
LPS conductor	-	1 kA 8/20 μs	-	-	-	-	100 kA 10/350 μs
SPD conductor	500 A 8/20 μs	-	20 kA 8/20 μs	40 kA 8/20 μs	60 kA 8/20 μs	80 kA 8/20 μs	100 kA 8/20 μs
LPS and SPD conductors	-	1 kA 8/20 μs	-	-	-	-	100 kA 10/350 μs

**Table 9:** Test results of $I_{tc}$.

Surge Current 8/20 μs	Number of Surge Tests	Count Results	Test Results
250 A (positive)	10	X (no count)	Pass
250 A (negative)	10	X (no count)	Pass
400 A (positive)	10	X (no count)	Pass
400 A (negative)	10	X (no count)	Pass
500 A (positive)	10	O (10 times)	Pass
500 A (negative)	10	O (10 times)	Pass

**Table 10:** Test results of the predictive lifetime of the SPD due to lightning surge currents.

Surge Current 8/20 μs	Surge Current Per Path	Decreased Predictive Lifetime (%)	Sample #1 (%)	Sample #2 (%)	Sample #3 (%)	Sample #4 (%)
1 kA	250 A	0.00	0	0	0	0
5 kA	1.25 kA	0.03	0.03	0.03	0.03	0.03
10 kA	2.5 kA	0.12	0.11	0.12	0.13	0.12
20 kA	5 kA	0.50	0.46	0.49	0.53	0.51
30 kA	7.5 kA	1.15	1.06	1.11	1.22	1.19
40 kA	10 kA	2.08	1.95	2.01	2.24	2.15
50 kA	12.5 kA	3.32	3.06	3.14	3.52	3.44
60 kA	15 kA	4.93	4.52	4.67	5.35	5.20
70 kA	17.5 kA	6.96	6.35	6.50	7.53	7.23
80 kA	20 kA	9.49	8.78	9.19	10.23	9.79
90 kA	22.5 kA	12.66	11.66	12.05	13.69	12.98
100 kA	25 kA	16.61	15.30	15.87	17.59	17.20

**Table 11:** Performance and cost comparison of SCDSs.

SCDS	Surge Count	Current Measurement	Polarity	SPD Predictive Lifetime	Cost	General Market
Proposed SCDS	O	O	X	O	Very low (tens of USD)	Applicable
Surge counter	O	X	X	X	Very low (tens of USD)	Applicable
SCDS with Rogowski coil	O	O	O	O	High (hundreds of USD)	Not applicable
SCDS with expensive CT	O	O	O	O	Very high(thousands of USD)	Not applicable
